# 2145. Economic Burden of Congenital Cytomegalovirus Infection in Commercially- and Medicaid-insured Patients in the United States

**DOI:** 10.1093/ofid/ofac492.1765

**Published:** 2022-12-15

**Authors:** John D Diaz-Decaro, Gail J Demmler-Harrison, Jessica R Marden, Annika Anderson, Sandeep Basnet, Katherine Gaburo, Danielle Peterson, Kosuke Kawai, Noam Kirson, Urvi Desai, Philip Buck

**Affiliations:** Moderna, Inc., Potomac, Maryland; Baylor College of Medicine, Bellaire, Texas; Analysis Group, Inc., Boston, Massachusetts; Analysis Group, Inc., Boston, Massachusetts; Moderna, Inc., Potomac, Maryland; Analysis Group, Inc., Boston, Massachusetts; Analysis Group, Inc., Boston, Massachusetts; Moderna, Inc., Potomac, Maryland; Analysis Group, Inc., Boston, Massachusetts; Analysis Group, Inc., Boston, Massachusetts; Moderna, Inc., Potomac, Maryland

## Abstract

**Background:**

Congenital cytomegalovirus (cCMV) infection is the leading infectious cause of congenital birth defects. Approximately 20-25% of infants born with cCMV develop long-term health complications such as hearing loss, developmental issues, and microcephaly. Despite this, studies on the economic burden of cCMV are limited. The aim of this study is to assess the healthcare resource utilization (HRU) and cost burden among a sample of cCMV patients in the US using insurance claims data.

**Methods:**

This retrospective study utilized IBM Watson Health MarketScan® Commercial Claims and Encounters and Multi-State Medicaid data from January 1, 2010 to December 31, 2019. Separately for each payer population, patients were included in the cCMV cohort if their first diagnosis (index date) of cCMV (ICD-9: 771.1; ICD-10: P35.1) or CMV (ICD-9: 78.5; ICD-10: B25.x) was within 1 month of birth. The index date for non-cCMV controls was selected at random from all medical claims within 1 month of birth. cCMV patients were matched 1:1 to controls on demographics, insurance type, birth year, and index year. All patients were required to have ≥ 1 year of continuous health plan enrollment post-index (study period). HRU and costs in 2021 USD ($) were summarized over the study period. Costs for birth admissions were also described.

**Results:**

195 Commercial and 549 Medicaid matched pairs were included in the analyses. Mean ± SD age at first diagnosis was 8.4 ± 8.6 days and 5.9 ± 7.8 days for Commercial and Medicaid cases, respectively. Mean birth length of stay for Commercial and Medicaid cases was 24 days (vs. 5 for controls), with mean birth admission costs of $149,192 (vs. $17,996) and $49,885 (vs. $5,052), respectively. On average, cCMV patients had higher study period HRU and costs compared to controls (Table 1). Excess costs due to cCMV were estimated at $33,223 for Commercial and $9,748 for Medicaid.

**Figure:**
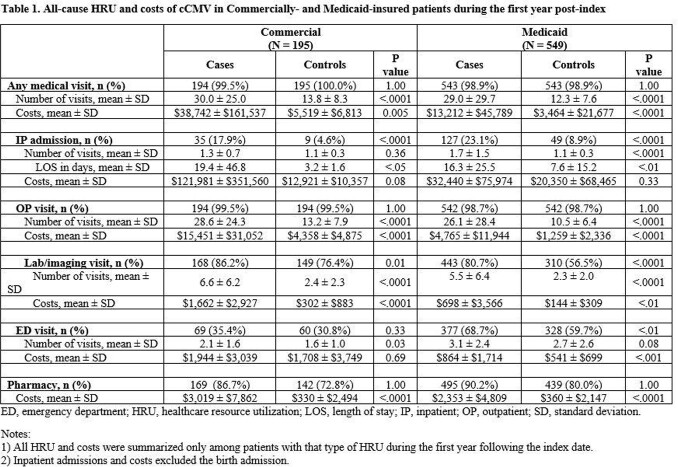

**Conclusion:**

cCMV patients have substantial HRU and costs during the 1-year post-diagnosis. While the majority of patients did not require hospitalization, inpatient care contributed substantially to the overall cost burden. Future studies should evaluate longer-term costs beyond the first year as well as the reasons underlying the high economic burden among cCMV patients.

**Disclosures:**

**John D. Diaz-Decaro, PhD, MS**, Moderna, Inc.: Salary|Moderna, Inc.: Stocks/Bonds **Gail J. Demmler-Harrison, MD**, Elsevier: Book royalties|Merck: Grant/Research Support|Microgen: Advisor/Consultant|Microgen: Grant/Research Support|Moderna: Advisor/Consultant|UpToDate Wolters Kluwer Health: Royalties **Jessica R. Marden, ScD, MPH**, Moderna, Inc.: Advisor/Consultant **Annika Anderson, MPH**, Moderna, Inc.: Advisor/Consultant **Sandeep Basnet, MD**, Moderna, Inc.: Salary|Moderna, Inc.: Stocks/Bonds **Katherine Gaburo, n/a**, Moderna, Inc.: Advisor/Consultant **Danielle Peterson, n/a**, Moderna, Inc.: Advisor/Consultant **Kosuke Kawai, ScD, MS**, Moderna, Inc.: Salary|Moderna, Inc.: Stocks/Bonds **Noam Kirson, PhD**, Moderna, Inc.: Advisor/Consultant **Urvi Desai, PhD**, Moderna, Inc.: Advisor/Consultant **Philip Buck, PhD, MPH**, Moderna, Inc.: Salary|Moderna, Inc.: Stocks/Bonds.

